# Recent advances in the intellectual property landscape of filamentous fungi

**DOI:** 10.1186/s40694-020-00106-z

**Published:** 2020-11-12

**Authors:** Silvia Hüttner, Anton Johansson, Paulo Gonçalves Teixeira, Puck Achterberg, Ramkumar B. Nair

**Affiliations:** 1 Mycorena AB, Kalkbruksgatan 4, 417 07 Gothenburg, Sweden; 2 Department of Biology and Biological Engineering, Division of Industrial Biotechnology, Chalmers University of Technology, 412 96 Gothenburg, Sweden; 3 Faculty of Applied Sciences, Delft University of Technology, Lorentzweg 1, 2628 CJ Delft, The Netherlands

**Keywords:** Intellectual property, Filamentous fungi, Patents, Biotechnology

## Abstract

For centuries, filamentous fungi have been used in the making of food and beverages, and for decades for the production of enzymes and pharmaceuticals. In the last decades, the intellectual property (IP) landscape for fungal technology has seen an ever increasing upward trend, introducing new and promising applications utilising fungi. In this review, we highlight fungi-related patent applications published during the last 5 years (2015–2020), identify the key players in each field, and analyse future trends. New developments in the field of fungal technology include the increased use of filamentous fungi as a food source (mycoprotein), using fungi as biodegradable materials, in wastewater treatment, in integrated biorefineries and as biological pest agents. Biotechnology companies in Europe and the US are currently leading when it comes to the number of patents in these areas, but Asian companies and research institutes, in particular in China, are becoming increasingly important players, for example in pesticide formulation and agricultural practices.

## Introduction

### Filamentous fungi

Fungi are eukaryotic organisms that have characteristics of both plants and animals but are placed in a distinct kingdom [1]. Fungi in general can be microscopic to macroscopic, and include unicellular organisms, such as yeasts, and multicellular organisms, such as filamentous fungi. Filamentous fungi grow as long, 2–10 μm thin filaments (hyphae) into intricate network structures (mycelium) that are observable to the naked eye and can grow to centimetre- to metre-scale. Filamentous fungi represent an incredibly rich and diverse group of species, with tens of thousands of fungal strains that have been identified, characterised, utilised and modified to this day. There is significant growth in the number of catalogued fungal strains, with an estimated 95% of fungal species estimated to be undescribed yet [2], and the scientific community is building an increasingly vast body of knowledge around these complex and valuable organisms. The large and growing interest in filamentous fungi mostly stems from the fact that their metabolic processes can be used to produce and refine a wide range of products and solutions [3] that create value for science, industries, and consumers.

Filamentous fungi, along with other microorganisms such as bacteria, yeast and algae, have been proven to be very useful for industrial applications [4]. Historically, their metabolites have created massive values in areas ranging from pharmaceuticals [5] to cosmetics [6] and commodity chemicals [7], and the production of these metabolites has had significant impact across the entire biotechnology spectrum. Starting with breakthrough developments of antibiotics in the early twentieth century, filamentous fungi have since then been used for applications that include producing biological control agents, enzymes, alcoholic beverages, organic acids, food and feed [8, 9]. With the rapid developments in strain discovery, strain engineering and industrial product development, it is certain that filamentous fungi will continue to be a key contributor in the creation of a biotechnology-based future.

### Filamentous fungi and intellectual property rights

As a result of these breakthrough solutions taking shape, activity in the intellectual property (IP) space is increasing as well. As the value and potential of filamentous fungi become more and more established and recognised, efforts to capture and control that value through the use of intellectual property rights (IPRs) have intensified across the globe [10]. Patents are arguably the most important IPRs for protection solutions, and the landscape unveils a growing body of patents surrounding novel processes and products related to filamentous fungi. This is indeed a strong indicator that international markets are realising the great potential that these organisms have, and that key industry players are doing what they can to secure value creation and value capturing through the use of IPRs.

Protecting filamentous fungi and other microorganisms with patents and other IPRs is challenging for two reasons. The first reason is that inventions relating to biological material are, by their nature, much more difficult to disclose than inventions in other areas. Patent law requires that a patentable invention be disclosed in a manner elaborate and clear enough so that a person skilled in the art can carry it out. With inventions relating to microorganisms, such disclosure is usually impossible. The Budapest Treaty, first signed in 1977 and entered into force in 1980, attempts to circumvent this problem by allowing instead for depositing samples of biological material to recognised institutions [11, 12]. Thus, all patenting procedures of new and modified strains of microorganisms, including filamentous and yeast-type fungi, follow the provisions of the treaty. The second reason is that much of the legislation around patenting of microorganisms is highly bureaucratic and difficult to comply with [13]. An example can be found in the Trade-Related Aspects of Intellectual Property Rights (TRIPS) agreement, whose provisions are well-known among industry players as very challenging to understand and live up to. A range of other issues have been created as a result of the ratification of TRIPS, all of which deserve analysis and discussion, but for the scope of this report it is sufficient to mention that many challenges are present.

Clearly, protecting technology surrounding filamentous fungi with patents and other types of IPRs is not an entirely easy ordeal. To truly realise and capture the great potential value of filamentous fungi, these challenges need to be thoroughly understood and taken care of. Related to this fact is that any industrial player should be mindful and attentive of past and current trends within the patent landscape of filamentous fungi. Despite the clear importance of filamentous fungi in developing ground-breaking biotechnology, efforts to summarise the critical and current IPR trends around filamentous fungi is lacking. This report therefore sets out to provide a thorough analysis of the recent patent landscape surrounding filamentous fungi and its various (industrial) applications. In addition, we seek to identify the key global players in the most important markets, and to make predictions about developments within this technological area for the coming years.

## Patent landscape

**Fig. 1:**
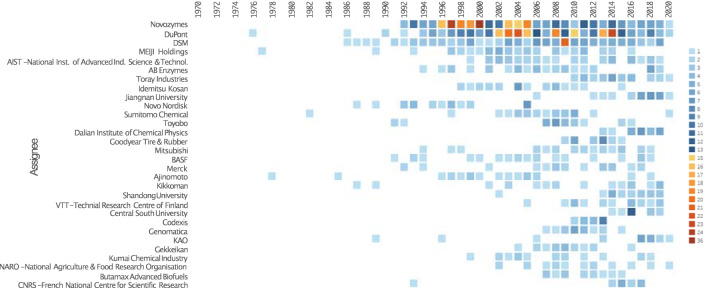
Heatmap of the top 30 organisations and their numbers of patent inventions (families) over time in the field of fungal technologies (for search terms, see “Methods” section)

**Fig. 2:**
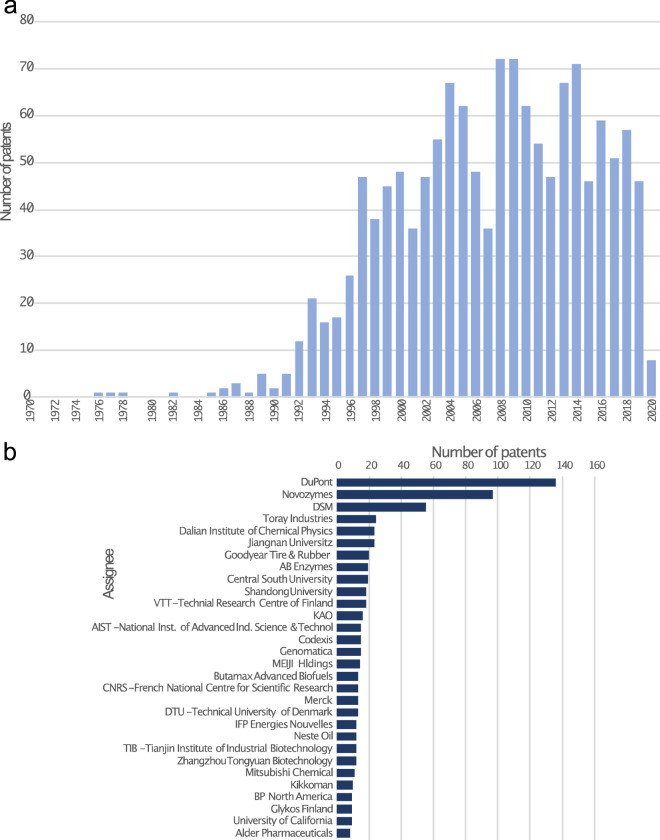
Overview of the development of the fungal technology patent landscape. **a** Increase of patents regarding fungi-related technologies and products over time (left). **b** Top 30 organisations and the numbers of patent inventions (families) in the field of fungal technologies with a publication date after 2015-01-01

Before analysing more specific fields of application, we will discuss the general patent landscape regarding filamentous fungi. First of all, it can be clearly seen that there has been a considerable increase in publications of patent families discussing filamentous fungi starting in 1990 (Figs. 1 and 2a). For the patent search and analysis the software Orbit Intelligence (Questel) was used (details in “Methods” section).

The top 5 players in terms of numbers of patents in the field of fungal technologies over the last 30 years were Novozymes (311), Dupont (289) incl. Danisco & Genencor, DSM (133), Meiji Holdings (44), and AIST (38)—International Institute of Advanced Industrial Science Technology. All are established biotechnology institutions that provide biological solutions for a variety of applications. The major technology areas over the same time frame were the following: 37.6% biotechnology, 15.0% basic chemical materials, 10.9% food chemistry. The majority of the patents categorised as biotechnology were found within the subgroup of enzymes and microorganisms.

During the last 5 years the distribution of patent classification in the field has not changed much, with 36.8% of patent applications being classified as belonging to the domain of biotechnology, while 15.7% were classified as basic materials chemistry, and 10% as food chemistry. The biggest observable trend concerned the countries of origin of patent applications. Here, an increase in patent families originating from Asian countries (mostly China and Japan) was noticeable. The key players of the last five years were: Dupont (137) incl. Danisco & Genencor, Novozymes (99), DSM (57), Toray Industries (24) and Dalian Institute of Chemical Physics (23) (Fig. 2b).

In the following sections, we describe the patent landscape in seven different subfields. Selected patents do not represent a complete list of published inventions, but instead were chosen to give an overview of the trends in the field and highlight important developments.

### Food and feed

Fungi have been used all over the world for millennia to produce various fermented foods and beverages [14, 15]. Traditional fermentation processes involving fungi and yeast are for example used to create soy sauce, miso, tempeh, “mould-cheeses” and beverages like beer, wine and spirits (Table 1). Mushrooms, the fruiting bodies of fungi, are of course also an important food, with the global mushroom market estimated to be worth over USD 42 billion [16]. However, in this study we did not include applications and inventions related specifically to mushrooms.

**Table 1:** Overview of different fungal species in traditional food applications. Adapted from Hyde et al. [15]

Application	Product	Fungal species
Beverages	Beer, rum, wine	*Saccharomyces cerevisae*
	Sake	*Aspergillus oryzae*
Cheeses	Roquefort, Blue cheese	*Penicillium roqueforti*
	Camembert, brie, soft ripened	*Penicillium camemberti*
	Reblochon	*Geotrichum candidum*
Other food products	Ang-Kak	*Monascus purpureus*
	Doenjang	*Aspergillus oryzae*
	Miso	*Aspergillus oryzae*, *A. sojae*
	Hamanatto	*Aspergillus oryzae*
	Oncom	*Neurospora intermedia*, *Rhizopus oligosporus*
	Shoyu (soy sauce)	*Aspergillus oryzae*, *A. sojae*
	Tempeh	*Rhizopus oligosporus*
	Viili	*Geotrichum candidum*
	Salami	*Penicillium nalgiovense*

Because of the many challenges related to global supply and demand for food, biotechnology has become an increasingly important solution for creating ways to feed the world. More recently, during the last decades, different microorganisms such as bacteria, yeast and algae have been heavily researched for their ability to create various types of biomass and compounds that can be used in production of food and animal feed [17, 18], with filamentous fungi being no exception, as they can produce a variety of useful food ingredients like enzymes, fatty acids, flavouring, organic acids, pigments and vitamins (Table 2).

**Table 2:** Overview of some metabolites obtained from filamentous fungi and yeasts. Adapted from Copetti [18]

Ingredient	Compound	Producing fungus	Application
Enzymes	Amylase	*Aspergillus niger*, *A. oryzae*	Production of glucose syrup, bread improvement, etc.
	Invertase	*Saccharomyces cerevisiae*, *S. uvarium*	Soft-centred candies, artificial honeys, confectioneries, liqueurs, etc.
	Galactosidase	*Mortierella vinaceae*	Beet sugar refining
	Lactase	*Aspergillus niger*, *A. oryzae*, *Kluyveromyces marxianus*, *K. fragilis*	Production of lactose-free milk and dairy products, upgrading cheese whey
	Pectins	*Aspergillus niger*, *Aspergillus* spp.	Juice clarification, improvement of grape juice yield, removing coffee mucilage, etc.
	Proteases	*Aspergillus oryzae*, *Aspergillus* spp.	Bread improvement, chill proofing of beer, milk coagulation, meat tenderization, etc.
Fatty acids	O3 and O6	*Mortierella alpina*, *Saccharomyces cerevisiae*, *Candida lipolytica*	Addition of polyunsaturated fat acids (bioactive compounds) to the composition of food and food products
Flavouring	Blue cheese flavour	*Penicillium roqueforti*	Impress blue cheese flavour in food products
	Bitter almond flavour	*Ischnoderma* spp.	Impress almond flavour in food products
	Roselike odor	*Saccharomyces* spp., *Kluyveromyces* spp.	General food flavouring
	Fruity, nutty, and fatty odor	*Candida lipolytica*, *Pichia ohmeri*	General food flavouring
Organic acids	Citric acid	*Aspergillus niger*, *Candida lipolytica*	Soft drinks, jams, jellies, candies, frozen fruits, dairy products, wine, etc.
	Gluconic acid	*Aspergillus niger*	Cleansing milk, beer and soft drinks bottles, baking products, etc.
Pigments and vitamins	Beta-carotene	*Blakeslea trispora*	Orange-red food colorants, vitamin A precursor and antioxidant
	Lycopene	*Blakeslea trispora*	Red food colorant and bioactive compound
	*Monascus* pigments	*Monascus* spp.	Spice and yellow, orange and red food colorants in Asia, meet preservatives
	Natural red	*Penicillium oxalicum*	Red food colorant
	Riboflavin	*Ashbya gossypii*	Yellow colorant and B2 vitamin

Within the area of food and feed applications, three main patenting sub-trends could be identified: production of food additives, using fungi as the main ingredient in new food products and using fungi for production of animal feed. All patents mentioned in this section can be found in Table 3.

**Table 3:** Overview of selected patent families associated with food and feed

Patent number (one of the family)	Countries	Assignee (application date, publication date)	Title
Food additives			
EP3063286	AT, AU, BE, CH, DE, DK, EP, ES, FR, GB, IE, JP, MX, NL, RU, SE, US, BR CA, CN, ID, IN	Conagen Inc. (2014, 2016)	Recombinant production of steviol glycosides
WO2019006244A1	AU, BR, CA, CN, EP, IN, KR, PCT WO	Conagen Inc. (2018, 2019)	Hydrolysis of steviol glycosides by beta-glucosidase
WO2018/056388	CN, JP, TW, PCT WO	Suntory (2017, 2019)	Method for production sesaminol or sesaminol glycoside
CN110746494	CN	Shenzhen Novojin Biotechnology (2019, 2020)	A group of special dietary proteins
EP3462915	AU, BR, CA, CN, EP, IN, JP, KR, MX, US	Cura Global Health (bvi) Ltd. (2016, 2019)	Process for forming iron enriched nutritional products
US20180021405	WO	Entia Biosciences Inc. (2017, 2018)	Nutritional approach to the control of anaemia, diabetes and other diseases or conditions and prevention of associated comorbid states with the use of ergothioneine
EP3182829	EP, WO	DSM IP Assets BV (2015, 2017)	Combination of glucose oxidases for improvements in baking
EP2869701	WO, AR	DSM IP Assets BV (2013, 2015)	Crisp baked products comprising xylanase
EP3302076	AU, EP, MX, US, WO	DSM IP Assets BV (2016, 2018)	Use of ice structuring protein afp19 expressed in filamentous fungal strains for preparing food
Traditional fermentation-related patents			
JP2016136843A	JP	Kikkoman Corp (2015, 2019)	Filamentous-fungus mutant in which hydrolase activity is improved
US20190159495	JP, US	Yamasa Corp (2018, 2019)	Method for short-time koji production using pre-cultured filamentous fungi
CN107494809	CN	Anhui Zhonghui Te (2017, 2017)	Black tea yeast starter and preparation method thereof
KR20180055255	KR	RDA—Korea Rural Development Administration (2016, 2018)	Method for manufacturing rice wine with reduced heat flavor, and rice wine manufactured by same
WO2019/067287	EP, US, PCT WO	DuPont Nutrition Biosciences ApS (2018, 2019)	Production of brewer's wort having increased fermentable sugars
JP2016123395	JP	Akita Konno Shoten (2015, 2018)	Method for producing tempeh fermentation product
JP2019129802	JP	Kikkoman Corp (2018, 2019)	Bean body structure solubilized product-containing food composition and process for producing same
Fungi as main ingredient			
EP0804086	Expired	Marlow Foods Limited (1995, 1997)	Texturised foodstuffs from gelled edible fungus and hydrocolloid mixture
EP3474685	GB, EP, TW, US	Marlow Foods Limited (2016, 2019)	Foodstuff
WO2018/002581	GB, TW, WO	Marlow Foods Limited (2017, 2018)	Edible fungus
WO2019/121697	EP, PCT WO	Lantmännen Energi (2018, 2019)	Process for industrial production of food-graded fungal biomass
US20190373934	EP, US, PCT WO	Emergy Inc. (2019, 2019)	Edible compositions including fungal mycelium protein
EP3464555	EP, WO	Mycorena AB (2017, 2019)	Process for edible filamentous fungi cultivation and its integration in conventional sugar to ethanol production
EP3080282	WO, CA, US, EP	Lantmännen Energi (2014, 2016)	Integration of first and second generation bioethanol processes
EP3209789	AU, CN, EP, US, WO	University of Strathclyde (2015, 2017)	Bioprocesses for co-production of ethanol and mycoproteins
WO2017/181085	US, AU, BR, CA, CN, EP, IN, JP, KR, MX, SG, WO	Mycotechnology Inc. (2017, 2017)	Methods for the production and use of myceliated high protein food compositions
WO2019046480A1	AU, CN, CA, WO, US, KR, EP, TW, IL, CO	Sustainable Bioproducts Inc. (2018, 2019)	Edible composition with filamentous fungi and bioreactor system for the cultivation thereof
US20200268031A1	WO, US	Sustainable Bioproducts Inc. (2020, 2020)	Food materials comprising filamentous fungal particles and membrane bioreactor design
Animal feed			
EP3385376	EP, PCT WO	AB Enzymes Oy (2017, 2018)	Fungal mannanases
WO2020/009964	EP, PCT WO	Dupont Nutrition Biosciences Aps (2019, 2020)	Xylanase-containing feed additives for cereal-based animal feed
CN106805002	CN	Zhejiang Baihui Biological Technology (2016, 2017)	Ferment fish guano for aquarium fishes
CN108450656	CN	Hunan Weigufang Biotechnology (2016, 2018)	Method for preparing functional feed additive by performing orientated fermentation on Chinese herbal medicine residues
CN109497259	CN	Zhengzhou Green Agriculture (2018, 2019)	Starter feed of sucking pig with disease-resistant function
CN108719578	CN	CAF—Chinese Academy of Forestry (2018, 2018)	Complete vegetarian pet food and preparation method thereof using beneficial fungus mixed culture technique

Looking at the patent landscape regarding food additives and filamentous fungi it could be seen that most patents relate to the production of enzymes or other compounds by either using a fungus as recombinant production host (i.e. inserting genes into its genome to produce new substances), or by using specific metabolites or enzymes native to the fungus. Filamentous fungi are highly important for their ability to selectively produce certain compounds of interest than can be used for areas such as nutritional supplementation, dyeing, and texture improvement. To name just a few examples of this, the patent landscape revealed new methods for producing sweeteners like steviol (EP3063286, WO2019006244A1) and other flavouring agents like sesaminol (WO2018/056388), as well as special dietary proteins (CN110746494) and a range of vitamins and minerals. Even some enriched nutritional products that include the whole fungi biomass have been developed, such as an iron supplement made with *Aspergillus oryzae* (EP3462915) or fungi that are enriched in vitamin D through UV irradiation (US20180021405). When looking at proteins and enzymes as food additives, the patents focus mostly on ones that improve textures and processability of materials, such as glucose oxidases for baking processes (EP3182829), xylanase to improve the storage of crisps (EP2869701) or ice structuring proteins to improve the freezing process of foods (EP3302076).

Even though fermentation with filamentous fungi has been used in food production for a long time, there are still improvements to be made. Accordingly, there was a surprisingly high number of new patents that relate to traditional food products and traditional food production processes. Improvements include hydrolases to increase soy sauce production (JP2016136843A), pre-cultures for faster koji production (US20190159495) and tea fermentation (CN107494809), removing off flavours in rice wine (KR20180055255), increased fermentable sugars in beer production (WO2019/067287) and better production of tempeh and other fermented bean-based foods (JP2016123395, JP2019129802).

One area that has seen a noticeable increase in patent applications in the last years is the production of food products that contain biomass or proteins of filamentous fungi as a or the main ingredient. Often, the application of the fungi here is called mycoprotein or, if the target area is animal feed, single cell protein (SCP). So far, there are only a handful of players in this field, but the trend certainly points towards an increase in this segment. The reasons for this are the high demands by consumers for more variety in the vegetarian and vegan product segment (e.g. to have an alternative to soy), the general increase of vegetarian and vegan products on the market, as well as the increased knowledge about the nutritional advantages and benefits of fungi-based protein. Mycoprotein is becoming an increasingly popular alternative to meat and even to meat alternatives like soy and pea, due to a very favourable amino acid profile and texture. We also identified examples of inventions related to further improving the attractiveness of filamentous fungi as a nutrient source by boosting the contents, ratios or availability of nutrients, as discussed below. One of the earliest patents in the area of mycoprotein were filed by Marlow Foods and describe production of what is now known as Quorn. Even though the original patents were filed in the 80ies (e.g. EP0804086), continuous improvements and inventions have been made, such as different ways to bind the proteins together (EP3474685) or reducing RNA levels in the final product (WO2018/002581). In recent years, the landscape has diversified, with more actors starting to file patents in the sector, in particular companies and organisations from Sweden, the UK and the US. They differ mostly in the use of different edible fungal species; while Quorn-related patents typically only use *Fusarium venenatum*, others use *Neurospora crassa* (WO2019/121697, US20190373934) or Zygomycete species (*Rhizopus oligosporus*, *R. oryzae*). We saw a clear indication that actors are moving to develop and protect more industrially focussed processes. Examples include patents that describe a possible integration of production of filamentous fungi for food (and feed) applications with bioethanol production (EP3464555, EP3080282, EP3209789). Furthermore, most patents describing mycoprotein production use a form of submerged fermentation (WO2017/181085), but some actors developed solid-state or surface-growth fermentation (WO2019046480A1, US20200268031A1).

Patents related to animal feed are mostly focussed on improving the digestibility of feed for the animal (IPC code A23K+). Most of these patents are protected in China and an increase has been seen in the last couple of years. Interestingly, many of the patents in this category can also have applications for human food, but do not necessarily mention this in the patent, nor is the patent categorised in human food applications.

Many examples of new patents related to animal feed, as will be expanded in more detail further below, were enzyme-related patents, such as the production of mannanases (EP3385376) and xylanases (WO2020/009964), as well as enzymes that break down fish guano for aquafeed (CN106805002). Other areas were related to preparing functional feed additives by using fungal fermentation (CN108450656, CN109497259). Pet food is another area where IP regarding filamentous fungi has increased in the last years, either as supplements produced by fungi, or as the whole fungi biomass to produce vegetarian pet food (CN108719578).

#### Key players

Historically, food-related applications of filamentous fungi have mainly been developed within the world of academia, however nowadays the landscape is dominated by established biotech companies which produce enzymes used in the food production. More specifically, of 324 identified relevant patent families in the field of food products, 38% were owned by the top 10 organisations. The key players from the last five years were DuPont (47 patents), DSM (16), AB Enzymes (13), Novozymes (11), Toray Industries (10). Marlow Foods had seven patents solely related to meat alternatives based on filamentous fungi. From a geographical perspective, the majority of patents were filed in China, closely followed by Europe, the US and Japan. Patents originating from Asia heavily focussed on traditional fermentation products, such as those of Kikkoman and Yasama, while Europe and the US focussed on patents related to the protein shift towards mycoprotein as a complete food source.

### Pharmaceuticals

The economic significance of filamentous fungi to the pharmaceutical industry cannot be overstated. Fungi have been widely exploited due to their capacity of producing a wide range of valuable compounds. Antibiotics, small molecules that inhibit the growth of microorganisms, in particular bacteria, are probably the best-studied fungal secondary metabolites [19]. However, although over a thousand antibiotic substances have been discovered in the past decades, less than around a hundred have been granted permission by regulatory agencies to be used in humans and animals, due to their toxic and adverse effects [20]. Most currently available antibiotics of fungal origin are found within the *Aspergillus* and *Penicillium* groups. Other fungi-produced pharmaceuticals include immunostimulants, anti-infectives, immunosuppressants and statins.

To identify the most important trends related to the production of antibiotics and other pharmaceuticals, we considered activities both within the healthcare sector, as well in sectors like agriculture and animal husbandry. The contents of recently published patents revealed for example that the growth inhibitory activity of certain antibiotics can be used against agricultural pests such as nematodes, arthropods and other parasites (EP3058939). Other compounds related to agricultural use of pharmaceuticals included biological fertilisers (CN107828699) and pesticides (WO2017188049), as well as fungal extracts that fight bee colony collapse disorder (US9474776B2).

In the healthcare sector, patents describing fungal technology were found for diverse applications, from biomaterials for the treatment of wounds (EP3165233) to products inhibiting the growth of oral bacteria in humans (WO201965124). Most new inventions, however, were not related to native fungal compounds, but to compounds produced in fungi as a production host, and are therefore related to the optimisation of cultivation techniques by genetically engineering the fungus (see also “Fungi cultivation methods” section). These included the production of antibodies (WO2019/175477, CN109575130, CN110256557) and glycan-based antibody-drug conjugates (EP3426288, US2017036233), the improvement of the production system itself (EP3004145), or the transformation of compounds through fungal fermentation (EP3442553) (Table 4).

**Table 4:** Overview of selected patent families related to pharmaceuticals

Patent number (one of the family)	Countries	Assignee (application date, publication date)	Title
EP3058939	JP, WO	NAI Inc. (2014, 2016)	Antiparasitic agent
CN107828699	CN	Weifang Huabin Biotechnology (2017, 2018)	Agricultural composite microbial agent and preparation method of same
WO2017188049	AR, CN, JP, KR, TW, WO	Kumai Chemical Industry (2017, 2019)	Microbial pesticide formulation composition, method for producing same, and method of using the same
US9474776B2	US, EP, AU, WO, CA, DK, ES	Paul Edward Stamets (2015, 2016)	Integrative fungal solutions for protecting bees
WO201965124	EP, PCT WO	Ikeda Shokken (2018, 2019)	Composition to inhibit proliferation of oral bacteria
EP3426288	US, AU, CA, CN, EP, IL, JP, KR, MX, SG, TW	Alder Biopharmaceuticals Inc. (2017, 2019)	Anti-pacap antibodies and uses thereof
US20170362338	US	Merck (2017, 2017)	Glycan-based antibody-drug conjugates
EP3442553	EP	CNRS—French National Centre for Scientific Research (2016, 2019)	Use of a *Withania* extract for the treatment of neuromuscular disease
EP3004145	US	DTU—Technical University of Denmark (2014, 2016)	Genetically modified filamentous fungi and uses thereof
WO2019/175477	EP, PCT WO	VTT—Technical Research Centre of Finland (2019, 2019)	A subunit vaccine against porcine post-weaning diarrhoea
CN109575130	CN	Aituojin Bio Pharmaceutical (2018, 2019)	Monoclonal antibody for detecting hpv18 e7 protein as well as preparation and application of monoclonal antibody
CN110256557	CN	Northeastern University of China (2019, 2019)	Anti-bap31 single-domain antibody and application thereof
EP3165233	EP	Latvijas University (2015, 2017)	Biomaterial for treatment of acute and chronic skin wounds

#### Key players

Overall, large organisations were not as prevalent in the pharmaceuticals field compared to other fields in this study. In the performed patent search, 23% of patents could be assigned to the top 10 organisations, the most prominent being Merck, Alder, DuPont, CNRS and Novozymes. While most of these are established biotech/pharmaceutical companies, a lot of IP was generated by universities and research centres such as CNRS and Shandong University, both of which are also among the top 10.

### (Bulk) chemicals

The production of bulk chemicals—such as low molecular weight organic acids—by filamentous fungi has attracted considerable attention due to their industrial applications. The demand for sustainable alternatives to petroleum as a source of fuel and a precursor for chemicals is high and has driven advancements in synthetic biology, genetic engineering and microbiology. Filamentous fungi are natural producers of a variety of molecules with potential applications. In addition, some species are capable of a high production of organic acids (e.g. citric acid, AU2008223787B2, US20150232892A1), which are often used as food additives and cosmetic ingredients. Organic acids are fully biodegradable molecules and can also be used as chemical intermediates for the production of biodegradable polymers. Furthermore, filamentous fungi are used to produce dyes, artificial aromas and flavourings. Chemically speaking, these include a wide range of aromatic molecules, polypeptides, oligo- and polysaccharides and phenolic compounds (US9637763, EP2754716, CN107987183). An interesting feature of the literature around fungi-based production of organic acids, and also of the patents covering the same, is that it is often sharply focused on the ability to produce individual compounds, and not so much on the general production processes of the fungi.

The wide variety of applications of organic acids was well reflected in the patenting landscape of filamentous fungi. Several patents disclosed novel methods for production and uses of C4-dicarboxylic acids (WO2018051837A1, US2013288321, EP2473600B1, US20150104543A1) which have the potential to create commercial value in several ways. Other patents disclosed fungi-based methods for production of oxalyl-CoA, glyoxylate and glycolic acid (WO2019020870A, US9783809B2) and fumaric acid (WO2017110970A1). We detected an increase in patents related to fungal production of itaconic acid over the last years. This acid has gained recent interest as a future bio-based platform chemical [21, 22] and efforts are being made to increase space–time yield and the fungal production system (EP2183367B1, WO2014142647A1, WO2017074533A1, WO2018037123A1, US10443077B2, CN105274153A). Other developments lie in the integration of different production processes and the use of low-cost raw materials (US20190360004A1). All patents mentioned in this section can be found in Table 5.

**Table 5:** Overview of selected patent families associated with (bulk) chemicals production

Patent number (one of the family)	Countries	Assignee (application date, publication date)	Title
AU2008223787B2	WO, MY, AR	Adcuram Nutrition Holding GmbH (2008, 2013)	Process for the preparation of citric acid employing filamentous fungi in a culture medium comprising glycerol
US20150232892A1	US	Battelle Memorial Institute Inc. (2015, 2015)	Enhanced citric acid production in *Aspergillus* with inactivated asparagine-linked glycosylation protein 3 (alg3), and/or increased laea expression
US9637763	AU, CH, DE, EP, GB, IE, JP, KR, MX, NL, NZ, RU, US, ZA, BR, CA, CN, IN	Rho Renewables Inc. (2012, 2017)	Recombinant production systems for aromatic molecules
EP2754716	CH, CN, DE, EP, FR, GB, IT, JP, US, IN	Shonan Technology Center Inc. (2012, 2015)	Method for producing useful metabolite from filamentous fungus
CN107987183	CN	Zhejiang University of Technology (2017, 2018)	Method for chitosan oligosaccharide extraction from filamentous fungi
WO2018051837A1	CN, JP, US, VN	KAO (2017, 2018)	Mutant filamentous fungus and method for producing C4-dicarboxylic acid using same
US2013288321	DK, MX, WO, US	Novozymes Inc. (2013, 2013 abandoned)	Methods for improved C4-dicarboxylic acid production in filamentous fungi
EP2473600B1	DK, MX, WO,	Novozymes Inc. (2010, 2016)	Methods for improving malic acid production in filamentous fungi
WO2019020870A	US, WO, EP	VTT—Technical Research Centre of Finland (2018, 2019)	Improved production of oxalyl-CoA, glyoxylate and/or glycolic acid
US9783809B2	FI, EP, US, WO, DK	VTT—Technical Research Centre of Finland (2012, 2017)	Eukaryotic cell and method for producing glycolic acid
WO2016193540A1	WO	VTT—Technical Research Centre of Finland (2016, 2016)	Direct conversion of sugars to glycolic acid
WO2017110970A1	CN, JP, WO, US	KAO (2016, 2017)	Method for producing organic acid
US20150104543A1	WO	DSM IP Assets BV (2014, 2015)	Organic acid production by fungal cells
EP2183367B1	EP, WP, US	Dutch DNA Biotech BV (2008, 2019)	Production of itaconic acid
WO2014142647A1	WO	Wageningen University (2013, 2014)	Fungal strains with improved citric acid and itaconic acid production
WO2017074533A1	WO	Battelle Memorial Institute (2016, 2017)	Enhanced itaconic acid production in *Aspergillus* with increased laea expression
WO2018037123A1	WO, EP, CN, US	Lesaffre Et Compagnie (2017, 2018)	Improved production of itaconic acid
US10443077B2	CN, EP, WO, US	DSM IP Assets BV (2016, 2019)	Fermentation process for producing itaconic acid under nitrogen free conditions
CN105274153A	CN	Jiangnan University (2015, 2016)	Method for increasing yield of itaconic acid produced by fermentation of *Aspergillus terreus*
US20190360004A1	DK, US, CA, WO, CN, BR, EP, ES	DSM IP Assets BV (2019, 2019)	Integrated process for coproducing alcohol and organic acid from lignocellulosic material

#### Key players

As with other trends described, major biotechnological industry players were prominent in this field as well, such as Novozymes and DSM. There were, however, organisations who are less active in other use areas of filamentous fungi that were present within this patenting space. Examples are companies such as RHO Renewables, KAO and Shonan Technology Center. On the academic side, we saw a strong presence of biology-oriented research institutions such as the Technical Research Centre of Finland (VTT).

### Enzymes

The advances in fungi and enzyme research have had fundamental implications for industrial biotechnology [23]. Filamentous fungi are natural producers of a variety of different enzymes and are excellent at secreting them into the culture medium. For that reason, they are often successfully used as a chassis for the development of recombinant production strains. Again, this area is indicative of the incredible versatility and flexibility that can be obtained by using filamentous fungi for the production of various compounds of interest, and we saw a very wide range of topics and inventions covered in the patent landscape of fungi-based enzyme production (Table 6).

**Table 6:** Overview of selected patent families associated with enzymes

Patent number (one of the family)	Countries	Assignee (application date, publication date)	Title
EP1799816B1	JP, EP, WO, CA, CN, MX, TA, MA	AB Enzymes Oy (2005, 2015)	Novel laccase enzyme and use thereof
WO2012023021A1	CN, WO, BR, DE	Council of Scientific & Industrial Research (2010, 2012)	Method for obtaining laccase enzyme from *Arthrographis* sp.
EP3099794	US, AR, AU, BR, CA, CN, EP, KR	DuPont Nutrition Biosciences ApS (2014, 2016)	Compositions and methods comprising a xylanase enzyme variant
EP2766471	AT, AU, CA, CN, CZ, DE, DK, EP, ES, FI, FR, GB, HR, HU, IN, IT, NL, NO, PL, PT, RO, SE, SK, US, BR	IFP Energies Nouvelles IFPEN (2011, 2017)	Process for the continuous production of cellulases by a filamentous fungus using a carbon substrate obtained from an acid pretreatment
WO2019122520A1	WO	AB Enzymes Oy (2018, 2019)	Variants of fungal cellulase
WO2019219804A1	WO	DSM IP Assets BV (2019, 2019)	Process for producing a polypeptide
WO2016090474A1	WO	Concordia University (2015, 2016)	Novel cell wall deconstruction enzymes of *Chaetomium olivicolor*, *Acremonium thermophilum*, and *Myceliophthora hinnulea*, and uses thereof
EP2906692	WO, MX	Danisco—Dupont (2013, 2015)	Method of using alpha-amylase from *Talaromyces emersonii* for saccharification
EP3385376	EP, WO	AB Enzymes Oy (2017, *2018*)	Fungal mannanases
WO2016142536A1	EP, US, WO, BR, CN	Genencor International BV (2016, 2016)	Enzymatic activity of lytic polysaccharide monooxygenase

Many disclosed inventions related to the production of specific enzymes, in particular lignocellulolytic enzymes, and related compounds of interest, such as laccases (EP1799816, WO2012023021A1) xylanases (EP3099794), phytases, cellulases (EP2766471, WO2019122520A1, WO2019219804A1, WO2016090474A1), amylases (EP2906692), mannanases (EP3385376) and lytic polysaccharide monooxygenases (LPMOs; WO2016142536A1) to give a few examples. As would be expected, these patents have diverse application areas, and there are clear connections to industries ranging from pharma to food to pulp and paper. Competition between actors producing these enzymes can be high, as the patent landscape revealed several patent disputes and invalidity actions between some of the strongest companies working within the field. This is perhaps not entirely surprising, as this part of the market space is inhabited by organisations with high levels of IP sophistication.

The main fungal production hosts have not changed much in the last decades, and were also in the selected patents mostly limited to *Trichoderma reesei* and *Aspergillus* species.

In the area of processes for enzyme production, patents were heavily focused on the general ability and potential of filamentous fungi to produce enzymes for a vast variety of purposes. The patents related to this field will be discussed in more detail in “Fungi cultivation methods” section.

#### Key players

Among the most prominent developers of fungi-based solutions for enzyme production is Novozymes, a leading Denmark-based biotechnology group. AB Enzymes stands out as a rising actor within the field, having filed several significant applications during the last few years. Other renowned names from the biotechnology space, such as Dupont, DSM, Codexis and Toray Industries, appear in the records as well. Academia is of course also well represented, with reputed organisations such as the Technical University of Denmark (DTU), India’s Council of Scientific & Industrial Research, VTT and Nagakoa University of Technology showing up repeatedly.

### Environmental technology

The domain of environmental technology holds a variety of inventions, with most patent inventions being associated with treatment of water (wastewater, sewage or sludge) or biological processes for separation. There has been a significant increase in inventions in this technical domain in recent years.

Interestingly, the area of using filamentous fungi in wastewater treatment is predominantly protected in China. In most of these inventions, fungi are integrated in the solution either as biomats or microbial sludge (US20190316077, CN206886941U, CN109370943, CN109092048, WO2018/014037). However, some patents were identified where enzymes from filamentous fungi are used, such as cellulases (EP3219797) and amylases, to break up compounds in the waste stream. Recent patents also included examples of degrading solid waste and transforming it into something with more value (CN108219887, EP2576213). The mentioned patents can be found in Table 7.

**Table 7:** Overview of selected patent families associated with environmental technology

Patent number (one of the family)	Countries	Assignee (application date, publication date)	Title
US20190316077	US, JP, SG, CN, CA, KR, BR, WO, EP, CN, AU	Sustainable Bioproducts Inc. (2016, 2019)	Filamentous fungal biomats, methods of their production and methods of their use
CN206886941U	CN	Jiangnan University (2017, 2018)	Sludge dewatering system is taken care of in online fermentation of filamentous fungi
CN109370943	CN	Hefei Huagai Biotechnology (2018, 2019)	A kind of microbial deodorant and preparation method thereof suitable for sewage treatment
CN109092048	CN	Wuhan Boyang Guangwu Technology (2018, 2018)	Microbiocidal deodorant
WO2018/014037	US, WO	University of California (2017, 2018)	Clarifying water and wastewater with fungal treatment/bioflocculation
EP3219797	JP, WO	RIKEN (2015, 2017)	Cellulase activator and method for saccharifying lignocellulosic biomass by using same
CN108219887	CN	Shandong Zhongrong Biotechnology (2017, 2018)	Method for producing biomass fuel blocks from sludge of sewage plant
EP2576213	AU, CA, CN, IL, IN, KR, MX, NZ, UA, ZA, BR, JP, SG, US, WO	Xyleco (2011, 2016)	Processing biomass

#### Key players

Most of the patents we analysed that fall into the area of biological water treatment were filed and protected in China (around 70%). The key players were Asia-based universities such as Central South University, Beijing University of Technology and Hohai University. Currently most patents are coming from research-based institutions, as it is still such a recent field. Another reason why the adoption by companies has not been as fast as in other segments is perhaps that it is not quite as straightforward to make money from wastewater treatment as it is through production and sales of chemicals, enzymes or pharmaceuticals. However, it is expected that in the future more companies will enter this landscape due to governmental incentives, penalties for environmental pollution and increasing value of clean production processes.

### Fungi cultivation methods

To keep up with the rapid developments within all aspects of filamentous fungi applications, the methods and tools for cultivation need to improve accordingly. It is clear from the patent landscape that companies and public actors are seeking new ways in which we grow and manage these interesting microorganisms. Different needs sprout these solutions, but the common goals to improve fungal cultivation have to do with increases in titre, rate or yield (TRY) of the process in question, as well as the efficient genetic modification of the production strain. Two main patenting sub-trends were identified: one that relates to novel cultivation techniques and molecular biology methods, and one that relates to physical equipment for improved production.

In the subcategory of novel cultivation techniques fall patents that deal with metabolic and genetic engineering, as well as the ways that fungi are cultivated, maintained, manipulated and stored. Examples include methods for continuous production of filamentous fungi (FR3071507), innovative solutions for producing fungi pellets of extraordinary density (WO2018/221482), novel biomat formation (KR20180117131A) and changing the viscosity phenotype of the organism (EP2673290). Furthermore, several novel processes have been disclosed in conjunction with the product patents described in the section below.

As mentioned above, inventions in the area are also focused on the ability and potential of filamentous fungi to produce enzymes for a vast variety of purposes. In this regard, many patents describe methods for modifying the fungal strains themselves at a molecular level. Examples include, but are not limited to, methods for continuous enzyme production (FR3071507), general genome editing applications using CRISPR-Cas9 (JP2018525983A, US20190194692A1), regulating gene expression in fungi and improving protein production or secretion (EP2576793B1, US9499826B2, US9512415, JPWO2017170917A1, CN109661402A, JP2018504936A), and using genetically modified filamentous fungi for producing various biosynthetic products (EP3004145).

In the area of cultivation equipment, the majority of patent-protected inventions relates to physical tools. These include new vessels for efficient fermentation of filamentous fungi (CN207062292), equipment for mechanical control of filamentous fungi ball size (CN108893254) and devices for maintaining fungal cultures (CN209002501U, CN109628275). In contrast to other microorganisms, filamentous fungi often form mycelial macrostructures during cultivation, and depending on the process this can be either a benefit or a challenge. Aside from aspiring to improve fungi cultivation in general, we interpret this apparent demand for improved hardware as an increasing interest in integrating fungal cultivation solutions into existing industrial infrastructure. All patents mentioned in this section can be found in Table 8.

**Table 8:** Overview of selected patent families associated with fungi cultivation method

Patent number (one of the family)	Countries	Assignee (application date, publication date)	Title
Novel cultivation techniques			
FR3071507	FR	AB7 Industry (2017, 2019)	Continuous production of filamentous fungi
WO2018/221482	US, WO, CN, JP	KAO (2018, 2020)	Method for producing filamentous fungal pellets
KR20180117131A	JP, CN, CA, WO, WP, CN, AU, TW, US	Sustainable Bioproducts Inc. (2017, 2018)	Biomat of filamentous fungi, its production method and its use
EP2673290	AU, BE, CN, DE, DK, EP, FI, GB, HK, JP, KR, NL, US, BR, CA	DuPont—Danisco US Inc. (2012, 2016)	Filamentous fungi having an altered viscosity phenotype
JP2018525983A	WO, CN, US, JP, EP	Danisco Dupont (2016, 2018)	Genome editing system and method of use
US20190194692A1	WO, US, EP	DSM IP Assets BV (2016, 2019)	A CRISPR-Cas system for a filamentous fungal host cell
EP2576793B1	US, EP, MX, ES, DK, WO, CN, CA, US	VTT—Technical Research Centre of Finland (2011, 2017)	Method for improved protein production in filamentous fungi
JPWO2017170917A1	CN, BR, US, CA, JP, AU, EP, WO, PH	Toray Industries Inc. (2017, 2018)	Protein production method
CN109661402A	CN, US, EP, BR, WO	Danisco Dupont (2017, 2019)	Protein production system of fungi
JP2018504936A	WO, KR, MX, JP, US, CN, EP, ZA	Danisco Dupont (2016, 2018)	Fungal strains and methods of use
US9499826B2	DK, EP, WO, US	VTT—Technical Research Centre of Finland (2010, 2016)	Production of proteins in filamentous fungi
US9512415B2	WO, DK, EP, US	VTT—Technical Research Centre of Finland (2011, 2016)	Method for protein production in filamentous fungi
EP3004145	WO	DTU—Technical University of Denmark (2014, 2016)	Genetically modified filamentous fungi and uses thereof
Cultivation equipment			
CN207062292	CN	Shandong Academy of Medical Sciences (2017, 2018)	A kind of filamentous fungi fermentation tank
CN108893254	CN	Yixing Boden Teco Industrial Equipment (2018, 2018)	Equipment for mechanically controlling sizes of filamentous fungi balls in fermenting process
CN209002501U	CN	Lingnan Normal University (2018, 2019)	Filamentous fungus mycelium culture device
CN109628275	CN	Shanghai Howsome Biotechnology (2019, 2019)	Improved small culture device for filamentous fungi and culture method thereof

#### Key players

There was not a particularly strong presence of the otherwise dominating actors in this particular field, even though they were present (e.g. Novozymes, Dupont, KAO). Rising in this landscape was AB7 Industry. However, most inventions relating to cultivation methods and equipment came from within academia, with organisations such as Shandong University of Medical Sciences, Lingnan Normal Universitym, VTT and Jiangnan University appearing repeatedly.

### Materials

A nascent field that is picking up momentum is the application of mycelium of filamentous fungi as a material. Pure, composite and cross-linked mycelium materials have been created, as well as polymers of mycelium and fungi-derived chitin–glucan films. A recent paper by Cerimi et al. [24] gives an overview of patents that cover the production or use of fungi as biomaterial between 2009 and 2018. Recent patents describe fungi biomaterials that include textiles (CN106758447B), insulations (US10407675B2, WO2018120823A1), packaging (CN108249037, US20180148682), leather-like materials (CN102329512A), composite materials (US20190090436A1, US20130202855, US20190390156A1, US20190284307A1), as well as methods on how to achieve these materials (US20200196541A1, WO2020115690A1). See Table 9 for more details.

**Table 9:** Overview of selected patent families associated with materials

Patent number (one of the family)	Countries	Assignee (application date, publication date)	Title
CN106758447B	CN	Beijing Zhongke Aobei Supersonic Wave Tech Research Institute (2016, 2018)	A method that prepares textile fabrics with ultrasonic waves
US10407675B2	US	Ecovative Design LLC (2017, 2017)	Method of fermenting mycelium composite material
WO2018120823A1	WO	Shenzen Technical University (2017, 2018)	Fungus-based biomass fireproof material using rice straw as main material and preparation method thereof
CN108249037	Revoked	Dongguan Hopeway Packaging Tech Co Ltd (2017, 2018)	Production method for organic packaging material
US20180148682	US, MX, WO	Mycoworks Inc. (2018, 2018)	Molding system for fungal structures
CN102329512A	CN	Ford (2011, 2016)	Production method for dehydrated mycelium elements for outfitting vehicle interiors
US20190090436A1	WO, US	Ecovative Design LLC (2018, 2019)	High density rigid molded body of composite mycological material
US20190390156A1	US, WO	Ecovative Design LLC (2019, 2019)	Open-cell mycelium foam and method of making same
US20190284307A1	CA, WO, US	Mycoworks Inc. (2019, 2019)	Deacetylation and crosslinking of chitin and chitosan in fungal materials and their composites for tunable properties
US20200196541A1	WO, US	Mycoworks Inc. (2019, 2020)	Mycelium growth bed with perforation layer and related method for creating a uniform sheet of mycelium from a solid-state medium
WO2020115690A1	WO	Mogu S.R.L. (2019, 2020)	Method of producing fungal mats and materials made therefrom

In most cases, the mycelium is grown into molds or as a biofilm, inactivated, dried and processed to become a stiff or elastic material. A variety of different fungal strains, mostly Basidiomycetes such as *Pleurotus ostreatus* and *Ganoderma lucidum*, were mentioned in the associated patents.

#### Key players

The recent patent landscape for fungi-based materials is still fairly small with only four main players, three of which are US-based companies: Ecovative (21 patents), Ford (9), Shenzhen Technical University (8) and Mycoworks Inc. (3).

## Future trends

Because of the constantly increasing demand for food, and the many challenges facing the food and feed industries, we believe that microbiological, and in particular fungal, solutions for the production of food products and nutrients will become ever more important in the future. While other microorganisms show great promise for rising up to the challenge as well, filamentous fungi have excellent prospects for assuming the role of the single most important source of microbial nutrition. This is related not only to the already high degree of consumer acceptance, but also to the robustness, (substrate) versatility, and speed of metabolic processes of filamentous fungi, as well as their favourable nutrient composition and naturally fibrous texture. The number of mycoprotein-related patents is expected to rise, from both small companies as well as multi-national organisations. But also inventions related to supplements, colours and aromas from fungal sources will further increase in the future. With the incredible versatility of filamentous fungi, we will continue to find novel application areas within the entire value chains of the food industries.

When looking at the pharma industry, there has been a clear decreasing trend among both academic and corporate organisations regarding antibiotics research. Novel antibiotic discovery has been stagnating for many years [25], and any newly created antibiotics are mere iterations of previous core concepts. The search for novel antibiotics is now considered a low-reward effort by research funding agencies. As for private players, the incentives for novel antibiotics research often do not match the effort required for development. Lack of interest comes not only from the high investment costs required, but also that, if discovered, novel antibiotics will only be prescribed as a last line of defence in order to prevent acquired resistance to these new drugs, generating rather low revenues for pharmaceutical companies. Instead, however, the recombinant production of pharmaceuticals in fungal production systems is steadily increasing, in particular with the rise of personalised medicine and antibody-focused treatments. With an efficient secretion system, and the ability to produce complex and glycosylated proteins and chemicals, fungi are well-suited as efficient production hosts, and genetic engineering tools will certainly make them even more efficient in the future.

The amount, as well as the types, of (bulk) chemicals produced by fungal fermentation will increase in the future, and IP will continually be generated even for traditional processes such as organic acid production. We see constant innovation with new products entering the market and enhanced microbes being used for improved production processes. The fermentation step to produce the chemicals is now in many cases a mature technology, and the main innovation lies in the bioengineering of the organisms.

Similar to chemicals, despite a long history of enzyme production with filamentous fungi, it is still an important topic of innovation today. Enzyme production has had a tremendous impact not only in technical industries, but also in healthcare and biochemical synthesis processes. The advancements of biotechnology in these fields pave the way towards lower production costs of enzymes, improved gene discovery methods and faster development of production hosts. The increased availability of optimised fungal enzyme production systems also increases their use in industries dealing with bulk and low value products. As a prime example, low enzyme production costs are a crucial part in making lignocellulose fermentation processes for biofuel and greener bulk chemical production (biorefineries) economically viable and competitive. Furthermore, with the accessibility of technologies for enzyme discovery and “bench-to-market” routines, we foresee the number of enzyme-related patents to steadily increase. This is expected to be populated mostly by large private players with access to high-throughput pipelines for enzyme discovery and testing.

The IP landscape in fungi-related environmental technology is also expected to grow in the near future as the need for efficient and benign waste(water) treatment becomes more and more crucial. Scarcity of water around the world will demand more advanced water and environmentally friendly treatment applications. Other important areas that demand solutions are more efficient resource use and protection of ecosystems from problematic eutrophication due to (over)use of fertilizers. Due to their versatile character filamentous fungi offer an excellent opportunity for solving these problems and can be regarded as crucial players in the circular economy [26]. We expect that more integrated processes, where filamentous fungi play a part in adding value to resource streams that are seen as waste, will be seen in upcoming patents.

Future developments in the field of fungal cultivation methods are more difficult to predict. The creation of IP for new equipment and techniques for cultivating microbes could logically follow the trend of fermentation technology developments in general. However, with the development of breakthrough gene engineering techniques and increasingly permissive regulation when it comes to GMOs, biotechnology as a field is shifting from a traditional fermentation-technology focus to a more microbe-engineering focus, so it would not be surprising if IP creation in the field of cultivation equipment will be decreasing over time, while the amount of patents being filed related to metabolic and genetic engineering will increase.

Finally, in the field of fungal biomaterials, we expect an increase in IP generation over the next decade, as the need and desire for renewable and biodegradable materials, that replace petroleum-based products, increases. Fungal biomaterials will shape the future of material sciences, design, fashion, architecture and other material applications.

## Conclusions

To summarise, the ability of filamentous fungi to produce a plethora of different compounds has been responsible for their past and continued industrial importance. The key trends that we identified regarding the patenting landscape in the last 5 years (2015–2020) are the use of filamentous fungi as a food source (mycoprotein) by a wider variety of players, the continued improvement of cultivation techniques mostly due to metabolic engineering and new genetic engineering techniques, and the use of filamentous fungi biomass as biodegradable alternatives to petroleum-based materials. Furthermore, the use of fungi in environmental technology is increasing, with China spearheading the inventions in this field, and filamentous fungi are also becoming an increasingly important part in pesticide formulation and agricultural practices. We will certainly see a further increase of IP being generated for filamentous fungi, since the need for green and sustainable technology in all areas of industry is increasing. With only a fraction of fungi characterised and still a lot to learn regarding their metabolism and production of secondary metabolites, the potential of these fascinating organisms has by far not yet reached its peak.

## Methods

Most patents were searched and analysed by using Orbit Intelligence (v.1.9.8), a software provided by Questel. The patents were searched, not by individual record, but by invention-based families (FamPat). In addition, they were searched without a restriction on countries or regarding their legal status, hence patents that were lapsed, expired or revoked would also show up in the search. Initially a set of defined key words in the title or claims served as a basic set of search parameters. With or without a specifically set time limit focusing either on patents with a publication date from 2015-01-01 to 28-02-2020 or patents dating as far back as possible in the Orbit Intelligence database. Next, within these basic searches, we looked at specific technology domains as provided by the software based on International Patent Classification (IPC) and Cooperative Patent Classification (CPC) codes. In addition, a combination of the provided technical domains at IPC/CPC code was sometimes used to get a better understanding. As an example, to search within the field of food chemistry in relation to animal feed, IPC: A23K+ was used.

The standard script that was used for the searches was:

("FILAMENTOUS" "FUNGI" OR "FILAMENTOUS" "FUNGUS" OR "FILAMENTOUS" "FUNGAL" OR "FILAMENTARY" "FUNGUS" OR "FILAMENTARY" "FUNGI" OR "MYCOPROTEIN" "FUNGI" OR "MYCOLOGICAL" "FUNGI" OR "MYCOLOGICAL" "FUNGUS" OR "MYCOLOGICAL" "FUNGAL" OR "ASPERGILLUS NIGER" OR "ASPERGILLUS ORYZAE" OR "RHIZOPUS OLIGOSPORUS" OR "RHIZOPUS ORYZAE" OR "FUSARIUM VENENATUM" OR "MYCOPROTEIN" "FUNGUS" OR "MYCOPROTEIN" "FUNGAL" OR "FUNGI-BASED BIOMASS" OR "FUNGAL BIOMASS")/CLMS/TI.

The statistical analysis was performed by the Orbit Intelligence software, however additional rules were added (if necessary) to group assignees if they were from the same mother company (e.g. Danisco & Genencor are grouped with DuPont), or from the same company/institute but applied their patents with slightly different assignee names. While these search and analysis techniques were used as the basis for the paper, it does not include all relevant patents in this field. There are additional patents that do not comply with the search words used but do describe a filamentous fungi application. Hence, the paper discusses some patents that fall outside the search. It should also be mentioned that the patents in “basic” search were not filtered regarding their relevance nor were they validated, hence the search could still include patents that have the right ‘words’, but do not have anything to do with the usage of filamentous fungi. Furthermore, especially within the technical domain of pharmaceuticals patents, some feature inventions protecting against filamentous fungi instead of using them.

## Data Availability

Not applicable.

## Abbreviations

IP: Intellectual property
IPR: Intellectual property rights
TRIPS: Trade-related aspects of intellectual property rights
SCP: Single cell protein
TRY: Titre, rate or yield
GMO: Genetically modified organism
